# Complete Genome Sequence of a Novel Deletion Porcine Reproductive and Respiratory Syndrome Virus Strain

**DOI:** 10.1128/genomeA.00486-13

**Published:** 2013-07-25

**Authors:** Junying Shen, Xing Yan, Jianming Dong, Yunbo Jiang, Liurong Fang, Shaobo Xiao, Huanchun Chen

**Affiliations:** State Key Laboratory of Agricultural Microbiology, Huazhong Agricultural University, Wuhan, China; College of Veterinary Medicine, Huazhong Agricultural University, Wuhan, China

## Abstract

Porcine reproductive and respiratory syndrome virus HZ-31 strain is different from any other previously sequenced porcine reproductive and respiratory syndrome virus strains. It contains a 59-amino acid (aa) discontinuous deletion in aa 467 to 474, aa 498 to 519, and aa 533 to 561 of *nsp2*. Here, we report the complete genome sequence of this novel Chinese virulent PRRSV variant.

## GENOME ANNOUNCEMENT

Porcine reproductive and respiratory syndrome (PRRS) is recognized as an important threat to the swine industry. Because of its special characteristics, such as reproductive failure and respiratory disease, PRRS has caused great economic losses since it was first reported in the late 1980s (1). PRRS virus (PRRSV) is an enveloped, positive-sense, single-stranded RNA virus belonging to the family *Arteriviridae* (2, 3). Since highly pathogenic PRRSV (hpPRRSV) first emerged as a mutant of PRRSV in China in 2006, the death of pigs has increased, causing high economic losses (4, 5). Unlike previously sequenced PRRSVs, genomic analyses have shown that hpPRRSV contains a 30-amino acid (aa) deletion (at aa 481 and 533 to 561) in its *nsp2*-coding region (6, 7).

The novel PRRSV strain HZ-31 was isolated from lung samples of pigs infected with PRRSV in central China in 2012. The complete genome sequence was generated with PCR using 14 pairs of oligonucleotide primers to amplify different regions of PRRSV. The PCR products were purified and cloned into pGEM-T Easy vector (Promega) and sequenced with ABI3730xl genome sequencer. The full-length sequence is 15,260 nucleotides. The terminal sequences were acquired by using a kit for rapid amplification of cDNA ends (RACE) (Clontech, Japan). The genome of HZ-31 exhibits 92.2%, 87.6%, and 95.2% sequence identity with PRRSV strains CH-1a, VR-2332, and JXA1, respectively, and 59.8% sequence identity with the European prototype Lelystad virus (LV).

HZ-31 contains a 59-aa discontinuous deletion in aa 467 to 474, aa 498 to 519, and aa 533 to 561 of *nsp2*, which is different from other hpPRRSV strains in China that do not have a deletion in aa 481 of *nsp2*; however, the site is deleted in other hpPRRSV strains, such as JXA1. Further amino acid analysis indicated that the predicted structural proteins GP2, M, and N of strain HZ-31 show 87.16%, 94.83%, and 95.93% amino acid identities with those of strain JXA1, respectively. As the most important neutralizing antigen of PRRSV, GP5 of HZ-31 shows 84.00% amino acid identity with that of JXA1. Phylogenetic analyses based on the complete genome show that HZ-31 is an independent branch between strain JXA1 and strain EM2007, another unique variant isolated in China (8). The fact that HZ-31 displays a larger deletion needs to be taken into consideration for further studies of PRRSV, and the genome data of HZ-31 will facilitate future research of the molecular pathogenesis and immunogenicity changes of PRRSV.

### Nucleotide sequence accession number.

The genome sequence of PRRSV strain HZ-31 has been deposited in GenBank under the accession no. KC445138.
